# A computational framework for the inference of protein complex remodeling from whole-proteome measurements

**DOI:** 10.1038/s41592-023-02011-w

**Published:** 2023-09-25

**Authors:** Marija Buljan, Amir Banaei-Esfahani, Peter Blattmann, Fabienne Meier-Abt, Wenguang Shao, Olga Vitek, Hua Tang, Ruedi Aebersold

**Affiliations:** 1 Department of Biology, Institute of Molecular Systems Biology, ETH Zurich, Zurich, Switzerland; 2 Present Address: EMPA, Swiss Federal Laboratories for Materials Science and Technology, St Gallen, Switzerland; 3 Department of Medical Oncology and Hematology, University and University Hospital Zurich, Zurich, Switzerland; 4 Institute of Medical Genetics, University of Zurich, Zurich, Switzerland; 5 Khoury College of Computer Sciences, Northeastern University, Boston, MA USA; 6 Department of Genetics, Stanford University School of Medicine, Stanford, CA USA; 7 Faculty of Science, University of Zurich, Zurich, Switzerland; 8 Swiss Institute of Bioinformatics (SIB), Lausanne, Switzerland; 9 Present Address: Department of Pathology and Molecular Pathology, University Hospital Zurich, Zurich, Switzerland; 10 Present Address: Idorsia Pharmaceuticals, Allschwil, Switzerland; 11 Present Address: State Key Laboratory of Microbial Metabolism, School of Life Science & Biotechnology, and Joint International Research Laboratory of Metabolic & Developmental Sciences, Shanghai Jiao Tong University, Shanghai 200240, China

**Keywords:** Computational biology and bioinformatics, Systems biology, Proteomics, Cancer

## Abstract

Protein complexes are responsible for the enactment of most cellular functions. For the protein complex to form and function, its subunits often need to be present at defined quantitative ratios. Typically, global changes in protein complex composition are assessed with experimental approaches that tend to be time consuming. Here, we have developed a computational algorithm for the detection of altered protein complexes based on the systematic assessment of subunit ratios from quantitative proteomic measurements. We applied it to measurements from breast cancer cell lines and patient biopsies and were able to identify strong remodeling of HDAC2 epigenetic complexes in more aggressive forms of cancer. The presented algorithm is available as an R package and enables the inference of changes in protein complex states by extracting functionally relevant information from bottom-up proteomic datasets.

## Abstract (web summary)

AlteredPQR is a software tool, available as an R package, to infer remodeling of protein functional modules from whole-cell or tissue lysate proteomic measurements.

## Main

Thanks to recent developments in mass spectrometry (MS) and related software tools^1,2^, it is becoming possible to measure protein quantities in cell lines, animal models and patient samples with a high degree of coverage, throughput and reproducibility^3^. MS-based proteomics is reaching speed, accuracy and consistency that make it suitable for clinical applications and biomarker assessments^4^. Notably, assessing not only individual protein quantities, but also the overall protein organization in the cell, could provide stronger associations to cellular and organism phenotypes. For establishing the cellular identity and response to stimuli, quantitative distribution and modular organization of cellular proteins are as important as the overall composition of its proteome. Core units of cellular functional modules are stable protein complexes. Due to the mechanism of complex assembly, individual subunits are often required to be expressed in defined quantitative relationships^5^. An implication of this is also that disease-associated deletion or inactivation of one complex member can directly influence quantities of other proteins in the same complex^6,7^, as well as the overall quantity and functional capacity of the complex. A growing body of evidence has demonstrated that proteomic data outperforms transcriptomic measurements in detecting correlated expression levels of protein complex members^8,9^. Moreover, protein expression correlation, when integrated with data on the composition of protein complexes, can provide a powerful means for defining stoichiometries of large complexes^10^ and detecting subunit exchange. A map of cellular functional modules is not static and formation and dissociation of functional modules can be essential for the enactment of condition- or disease-specific cellular processes; however, systematic probing of protein organization in the cell is time consuming and technologically challenging^11^.

Here we developed a software tool to infer remodeling of protein functional modules from whole-cell or tissue lysate proteomic measurements. This approach enables identification of disease-associated alterations in protein quantitative relationships (PQRs) and represents a new concept that can also be applied to biomarker studies. Changes in PQRs are expected to have stronger phenotypic effects when they disrupt finely tuned cellular processes, such as the formation of a protein complex or regulation of a signaling pathway. We made the method available as an R package called AlteredPQR (Supplementary software) and applied it to proteomic datasets for breast cancer (BC) cell lines and patient samples. We were able to identify individual protein pairs as well as whole protein complexes that have established roles in cancer and whose quantitative relationships were strongly altered in a subset of disease samples. Among others, this included formation of epigenetic regulator complexes centered on the Histone deacetylase 2 (HDAC2) protein in the more aggressive forms of BC. In addition, we found that significant alterations in PQRs were predictive of the activity of related cellular pathways, as measured from protein phosphorylation status and that the PQR status of several protein pairs in cancer cell lines was associated with their drug response. Of note, many of the disease proteins identified with this approach were not strong hits on the single protein level. AlteredPQR provides an easy to use, efficient workflow that expands the discovery potential from quantitative proteomics measurements without any additional experimental work.

## Results

### Workflow for detecting altered protein quantitative relationships from quantitative proteomic datasets

The AlteredPQR software tool is applicable to quantitative proteomic datasets generated with a wide range of available MS methodologies. Our aim was to design an approach that (1) can identify multiple outlier values in test samples; (2) is easily interpretable; and (3) is able to compare a priori biologically different samples, such as disease samples to corresponding healthy controls. For the latter, we use for instance the non-disease set to obtain an insight into the distribution of the reference PQRs, as well as the technical and biological variability in the measurements. Here, we focused only on protein pairs that are known or inferred to be associated through forming a protein complex. For this, we used a list of interacting proteins compiled from several publicly available databases and experimental datasets, including CORUM, Reactome and Interactome3D complex assignments and protein pairs found in multiple affinity-purification MS studies^12–15^ (Methods). It is important to note that the observed relative changes in protein expression levels do not indicate absolute protein quantities and do not point to stoichiometry ratios in protein complexes (Supplementary Notes). They rather indicate the overall change in protein quantitative ratios in the cell, which is known to have a stronger phenotypic relevance for proteins that are components of the same protein complex or members of the same signaling pathway. Steps of the algorithm are shown in Supplementary Fig. 1 (1) for an individual protein pair, median and median absolute deviation (MAD) of their PQRs (represented as a log ratio of the protein quantities) are estimated from the measurements in the reference samples; (2) individual disease samples (or other test samples) are analyzed to assess standardized distances from the reference distribution and determine if the PQR values represent outliers, which are identified using modified *z* score estimates (Methods); (3) significant *z* scores (>3.5) are summed up and a *P* value for each pair is calculated by using a background score distribution for random protein pairs in which proteins were randomly assigned reference sample measurements of the studied proteins; and (4) finally, to ensure non-redundancy in the final list of significant protein pairs, each protein that was contributing to the signal is represented with the pair that had the highest total score. The last step ensures that in cases where the abundance of a single protein is strongly up- or downregulated, this change is still represented with only one pair. Together, this allows for identification of easily interpretable shifts in PQRs and confident detection of multiple outliers in tested samples.

### Application of AlteredPQR method on BC cell line and patient data

To assess the performance of the method, we used a publicly available proteomic dataset of 41 BC cell lines composed of 24 basal and 17 luminal cell lines^9^. BC is a heterogeneous disease where patient classification and treatment decisions are primarily based on the immunohistochemistry assays that assess expression of estrogen (ER) and progesterone (PR) hormone receptors, as well as of HER2 tyrosine kinase receptor. Severity of the disease also differs among the subtypes; patients with luminal A (defined as ER- and PR-positive and with a low proliferation rate) tend to have the most favorable prognosis, whereas patients with triple negative (TN) (negative for all three receptors) lack evident drug targets and tend to experience aggressive disease progression (Supplementary Table 1).

We first compared the modified *z* score ratio values implemented here to Mahalanobis scores that were previously used for the identification of single protein pair outliers in the compendium of the same BC cell lines measured by MS-based proteomics^9^. There, measurements from all samples were assessed together to define a background distribution and identify single sample outliers. We compared outlier scores of the two metrices when these were calculated with a ‘reference free’ method on all cell lines together and when luminal BC cell lines were used as a reference set and outliers were searched for in basal cell lines. For the latter approach, population mean and covariance matrix in the Mahalanobis equation were calculated using only values for the luminal cell lines. Use of a prespecified reference set we introduce here provides more power for the identification of multiple samples in the test set whose values lie outside of the expected distribution in, for instance, reference non-disease samples. We found that the values of the two outlier measurements correlated well (*r* = 0.7) and that they had a substantial overlap of top hits (Supplementary Fig. 2a–g and Supplementary Table 2). Our motivation to implement the approach that uses modified *z* scores is based on (1) intuitive interpretation of results and quick assessment of the validity of identified outliers; (2) straightforward implementation of the nonparametric approach that is not sensitive to technical outliers in the reference set; and (3) predefined and widely accepted outlier threshold (absolute (Mi) > 3.5) that simplifies the definition of multiple outlier samples^16^. Of note, the 3.5 threshold can still be associated with >5% of false-positive outlier values, depending on the reference sample size^16^.

We next used measurements in luminal (hormone receptor-positive) BC cell lines as a reference set (we considered only ten luminal cell lines that were ERBB negative) and looked for significant outliers among the basal BC cell lines. Of note, the majority of TN cancers are of a basal origin and 22 out of the 24 basal cell lines used in the analysis were also TN. We found that by analyzing the altered PQRs, it was possible to obtain information on the plausible remodeling of protein interactions, which is not readily evident from studying individual protein quantities. Protein pairs with strongly altered PQRs in basal cell lines (Supplementary Table 2) included components of the CIN85 complex, which is important for cellular invasion and associates with aggressive BC phenotypes^17^ and were more strongly enriched in cancer related roles when compared to significant hits from a standard protein-level comparison (KEGG ‘Pathways in cancer’ was the most significantly enriched term, Benjamini–Hochberg (BH)-adjusted *P* value < 3.1 × 10^−5^, Fisher’s exact test; Supplementary Note 1 and Supplementary Fig. 3a–c). Additionally, we used data on drug sensitivity for the same cell lines^9^ to assess phenotypic associations of the identified altered PQRs. We found that PQRs of several protein pairs were predictive of a response to drugs that were previously discussed as candidates for TN BC treatments (Supplementary Note 1, Supplementary Table 2 and Supplementary Fig. 3d).

**Fig. 1:**
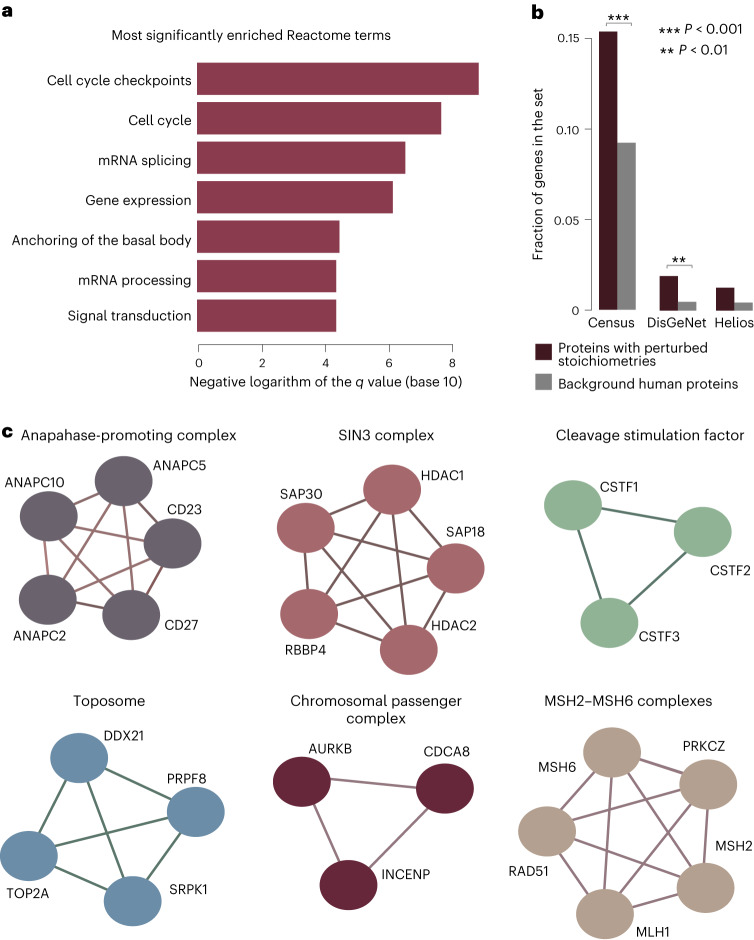
Inference of remodeled protein interactions in the BC patient samples. **a**, Reactome pathway terms that are significantly overrepresented among the proteins with altered PQRs when compared to all other analyzed proteins are shown. Bar-plot shows negative logarithms of *q* values for the significantly enriched Reactome terms, identified with a hypergeometric test and adjusted for multiple testing with FDR. **b**, Proteins with altered PQRs include a substantial number of known cancer drivers and proteins associated with BC (based on DisGeNET annotations and Helios predictions). Fraction of cancer-associated proteins in the set of proteins with altered PQRs is shown together with their fraction in the background set. *P* values were calculated with the two-sided chi-squared test (*P* < 5.5 × 10^−4^ and *P* < 4.4 × 10^−3^ for an enrichment in all Cancer Census Genes and in BC genes annotated in the DisGeNET database, respectively). **c**, Protein complexes for which the majority of the measured subunits had altered PQRs are shown. For CSTF and MSH2–MSH6 complexes, protein complex subunits were jointly upregulated (Supplementary Table 3) but their quantitative relationships were altered with respect to other interaction partners. Groups of proteins connected with edges in this panel show subunits of the same CORUM complex. Complex names correspond to CORUM annotations and edges indicate physical association within the same complex.

**Fig. 2:**
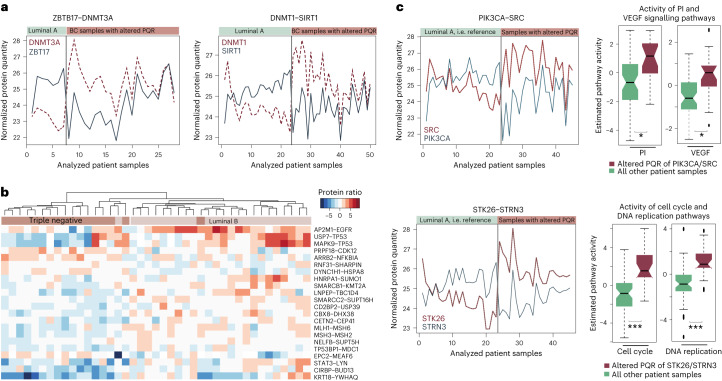
Examples of epigenetic and signaling interacting proteins that have altered PQRs in BC patient samples. **a**, Protein quantities for the two epigenetic protein pairs with the strongest signal are shown (ranked first and third in the overall AlteredPQR scores; all significant pairs are listed in Supplementary Table 3). The two pairs include DNA (cytosine-5)-methyltransferases 3A and 1 (DNMT3A and DNMT1) with their interacting proteins sirtuin 1 (SIRT1) and zinc finger and BTB domain containing 17 (ZBTB17). For both of these pairs, altered PQR is driven by expression changes of both proteins in the complex and marked with a strong shift in the relative protein quantities. Protein quantities measured in luminal A (LumA) BC samples (left, green) and protein quantities measured in all other BC samples (right, brown) are shown. **b**, Expression levels of a subset of protein pairs with altered PQRs significantly differed between the luminal B and basal samples. Protein pairs whose quantities most strongly differed between the two BC subtypes were identified with a Wilcoxon test (BH-adjusted *P* value < 0.05). Ratios of expression values for these pairs are shown. The significant instances included proteins with roles in DNA damage response, chromatin regulation and cell cycle. **c**, A shift in the relative quantities of signaling proteins can be coupled to phosphorylation changes in the related cellular pathways. Pathway activity in patient samples in which the protein pairs had altered PQRs were compared to their activity in all other measured samples (Supplementary Table 3) with a two-sided Mann–Whitney *U*-test and corrected for multiple testing with the BH method. Pathway activity scores are estimated from the phosphorylation status of all pathway proteins. Example pathways with the strong activity differences are shown and detailed description of the results is provided in Supplementary Note 2. PI, phosphoinositide 3 kinase pathway; VEGF, vascular endothelial growth factor. *denotes a *P* < 0.05 and ***P < 0.001.

To identify alterations in PQRs that are directly relevant in BC clinical manifestations, we further applied the AlteredPQR method to MS proteomic measurements from a cohort of 77 patients with BC and 3 healthy breast tissue samples^18^. This dataset included luminal A, luminal B, HER2 and basal patient samples (Supplementary Table 1). Patients with luminal B are hormone receptor positive (positive for the expression of ER and/or PR receptors), but with a higher proliferation rate than luminal A. We used the 23 luminal A samples as a reference set and searched for interacting protein pairs that showed altered PQRs in luminal B, HER2 or basal samples. The latter three tumor subtypes were represented with 24, 12 and 18 samples, respectively. On the molecular level, the luminal A subtype exhibits similarities with healthy tissue^19^. AlteredPQR identified 187 protein pairs with 318 proteins in total, whose PQRs in a subset of tested samples were outliers from their respective values in luminal A samples (adjusted *P* value for a comparison to a reference distribution composed from randomly sampled luminal A values < 0.1; Supplementary Table 3). To characterize functional categories associated with the identified proteins, we used their Reactome pathway annotations and compared them to the background of all analyzed proteins (Methods). Among others, this highlighted strong overrepresentation of gene expression, cell cycle and signal transduction functional categories in the set of proteins with altered PQRs (FDR-adjusted *q* < 4 × 10^−5^, hypergeometric test; Fig. 1a). Of note, only 21% (67) of the proteins with altered quantitative relationships were also among the top-most up or downregulated proteins in the same size set (Supplementary Table 3), thus indicating that the PQR analysis captured an additional layer of cellular regulation. In addition to being enriched in the processes that regulate cell identity, outlier protein pairs also contained a significant number of known cancer drivers (*P* < 5.5 × 10^−4^, chi-squared test for an enrichment in all Cancer Census Genes compared to a background set of other analyzed proteins and *P* < 4.4 × 10^−3^, chi-squared test for an enrichment specifically in BC genes annotated in the DisGeNET database; Fig. 1b). Furthermore, in accordance with general trends, CORUM protein complexes with the highest number of subunits among outlier protein pairs included those with roles in DNA replication, genome integrity and epigenetic regulation (Fig. 1c and Supplementary Table 3). As an illustration, 70% of the measured subunits of the SIN3 epigenetic regulator complex were strong outlier hits (Fig. 1c); proteins encoded by the *HDAC1, HDAC2, RBBP4, SAP18* and *SAP30* genes. Of note, the same proteins are also involved in other epigenetic regulatory assemblies apart from the SIN3 complex and are well known regulators of cell proliferation and cell cycle progression^20–22^. In a similar vein with these observations, two of the top three pairs with the most significant AlteredPQR values also included epigenetic regulators; DNA (cytosine-5)-methyltransferase proteins 3A and 1 (encoded by the *DNMT3A* and *DNMT1* genes). Relative protein quantities across samples for the respective pairs are depicted in Fig. 2a. Furthermore, differences between luminal B and basal samples further highlighted BC subtype-specific changes in epigenetic complexes (Fig. 2b and Supplementary Note 2). In addition, we explored phosphorylation profiles across the same patient samples^18^ and found that AlteredPQR scores for several signaling proteins strongly associated with activities of the associated downstream pathways (Fig. 2c, Supplementary Note 2 and Supplementary Table 3).

### Assembly of specific epigenetic complexes associates with more aggressive breast cancer

For protein subunits of the same complex, a substantial change in the correlation of their expression values between different sample groups can indicate an assembly or disassembly of the respective protein complexes^6^. We used the same BC patient proteomic measurements as above^18^ to explicitly address protein correlation changes between more aggressive basal BC samples and less aggressive luminal A samples. For this, we calculated pairwise correlations for all protein pairs with evidence they can form a protein complex (criteria as described above) separately in luminal A and separately in basal samples. We considered that a protein pair had a strong correlation shift between the two BC subtypes only when the pairs significantly correlated in one, but not in the other subtype (Pearson correlation *r* > 0.6 and BH-adjusted *P* value ≤ 0.05; the correlation threshold of 0.6 is used to capture moderate to strong positive relationships and *P* value was calculated using Pearson’s correlation coefficients). We also required the shift in the correlation values between the two BC subtypes to be clearly evident (absolute change in the Pearson *r* value > 0.6). In this way, we identified in total 464 protein pairs with a strong correlation shift (Supplementary Table 4) and 260 of these pairs correlated strongly in basal samples (*r* > 0.6), but did not correlate at all, or had a negative correlation, in luminal A samples.

**Fig. 3:**
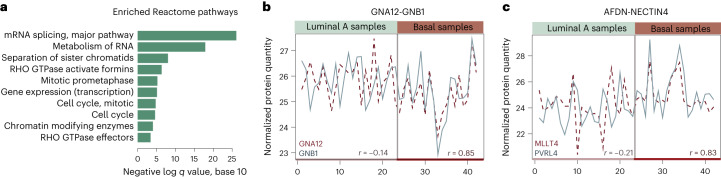
Protein complexes whose subunits have coordinated expression levels in more aggressive forms of BC. **a**, Reactome pathways overrepresented among protein complex subunits whose expression levels correlate strongly only in the more aggressive basal BC subtype are shown. The bar-plot indicates a functional enrichment for each term when proteins with a correlation gain in the basal BC samples were compared to all other proteins included in the analysis. Enrichment is calculated with the hypergeometric test and corrected for multiple testing with FDR. **b**,**c**, Examples of protein pairs with a notable correlation gain in basal BC samples. Strong alignment of the expression values of two Rho signaling proteins encoded by the *GNA12* and *GNB1* genes in more aggressive cancers (Pearson’s *r* = 0.85) could associate with a coordination of Rho signaling activities and cytoskeleton remodeling which is known to play a role in metastasis and invasion (**c**). Coordinated upregulation of proteins encoded by the *AFADIN* (*AFDN*, also *MML4*) and by the *NECTIN4* (also *PVRL4*) genes in a subset of more aggressive cancers (Pearson’s *r* = 0.83) is of interest as *NECTIN4* has been identified as a biomarker for BC metastasis and stem cell state (**c**). The encoded protein is involved in cell–cell adhesion, migration and proliferation and it organizes intercellular junctions together with the protein product of the *AFDN* gene, which has a role of a scaffold protein.

The latter 260 pairs included 338 proteins in total. These proteins were highly enriched in functional roles associated with the regulation of gene expression, cell cycle, chromatin modification and signaling (for this we used Reactome pathway analysis in a comparison to all other genes that were included in the analysis, that is were measured and are known to form complexes; *q* value < 5 × 10^−4^, hypergeometric test; Fig. 3a). In total, 59 (17%) of the proteins that correlated strongly only in basal samples were annotated as chromatin regulators (cataloged as ‘Chromatin modifying enzymes’ in Reactome or annotated with a Gene Ontology (GO) molecular function term ‘Chromatin binding’ or a GO biological process term ‘Chromatin remodeling’). Different complexes assembled by these proteins are known to play a crucial role in regulating cancer cell growth and driving cancer progression^23–26^. Instances in which both proteins had an annotated role in epigenetic regulation are listed in Table 1. Based on the manually curated protein complex annotations from the CORUM database^13^, eight of these proteins (encoded by the *HDAC2, CHD4, RBBP4, MTA1, MTA2, KDM1A, RCOR1* and *SIN3A* genes) can be found within the HDAC2 complex, five proteins (encoded by the *HDAC2, CHD4, RBBP4, MTA1* and *KDM1A* genes) are subunits of the nucleosome remodeling and deacetylation complex, whereas six are components of the related nucleosome remodeling and histone deacetylation (NuRD) complexes 1 and 2 (encoded by the *HDAC2, CHD4, RBBP4, MTA1, MTA2* and *MTA3* genes). In addition, eight of these proteins (encoded by the *HDAC2, RBBP4, ACTL6A, SMARCB1, SMARCC1, ARID1A, SAP30* and *SIN3A* genes) are subunits of the SIN3–ING1b complex. Some of the proteins have well-established roles in BC, such as protein encoded by the *CHD4* gene, which can promote BC growth and progression^27^, protein encoded by the *RBB4* gene, whose high expression levels associate with poor BC prognosis^28^ and protein encoded by the *HDAC2* gene, which is found overexpressed in different cancers and considered an important target in cancer therapy^29^ (Supplementary Note 3). Examples of two protein pairs that correlate highly in basal, but not in luminal A samples are shown in Fig. 3b,c. Overall, this analysis indicates substantial remodeling of regulatory complexes in the more aggressive forms of cancer and we find that this can be monitored from the whole-proteome measurements. The analysis of marked changes in correlation values is included in the AlteredPQR R package as a standalone function CorShift.

**Table 1:** Correlation changes among subunits of several epigenetic complexes associate with more aggressive forms of BC

Protein pair	Correlation in luminal A samples	Correlation in basal samples	*P* value for the correlation in basal samples	Adjusted *P* value for the correlation in basal samples
ARID1A–SMARCC1	0.2	0.82	0	0
ACTB–ERG	0.14	0.77	3×10^−4^	0.01
MTA1–SMARCA5	−0.21	0.76	2×10^−4^	0.01
MTA1–CHD4	0.09	0.73	4×10^−4^	0.01
HDAC2–MTA3	−0.2	0.72	6×10^−4^	0.01
SIN3A–RBBP4	−0.12	0.71	7×10^−4^	0.01
KDM1A–SMARCA5	−0.11	0.7	9×10^−4^	0.02
SIN3A–MTA2	0.04	0.7	9 ×10^−4^	0.02
RBBP4–CENPS	−0.44	0.79	0.0014	0.02
HDAC2–RBBP4	−0.03	0.68	0.0015	0.02
DMAP1–ACTL6A	−0.2	0.67	0.0016	0.02
HDAC2–MTA1	0	0.67	0.0016	0.02
HCFC1–SAP30	0.03	0.67	0.0017	0.02
DPY30–CHD4	−0.44	0.67	0.0018	0.03
TAF12–SMARCB1	0.01	0.77	0.002	0.03
ADNP–CBX3	−0.08	0.65	0.0025	0.03
HDAC2–RCOR1	−0.05	0.65	0.0028	0.03
USP22–SGF29	−0.34	0.65	0.0029	0.04
RBBP4–CHD4	−0.06	0.64	0.0032	0.04
SUPT7L–TAF10	0.01	0.69	0.0034	0.04
RBBP4–SMARCA5	−0.35	0.63	0.0039	0.04
RUVBL2–MEAF6	−0.16	0.62	0.0043	0.05
SMC3–SMARCA5	−0.08	0.62	0.0044	0.05
CCND1–ESR1	−0.01	0.69	0.0045	0.05
CHD4–ACTL6A	−0.37	0.62	0.0047	0.05
HCFC1–RUVBL1	−0.07	0.61	0.0052	0.05

Protein pairs where the two proteins can form a stable interaction and whose expression levels do not correlate in luminal A samples, but show a significant correlation in basal BC patient samples are listed. Only instances in which both proteins have an annotated role in epigenetic regulation are shown. Correlation gain is indicative of the complex formation in more aggressive forms of cancer. The shown *P* values were calculated with a two-sided correlation test implemented in the R function cor.test, using Pearson’s coefficients. They were adjusted for multiple testing with the BH method.

## Discussion

Jointly, these results demonstrate that altered PQRs and protein correlation shifts can provide a complementary view into the assembly and disassembly of both stable protein complexes and less-stable interaction modules. The observed trends indicate protein complex changes that strongly associate with BC clinical phenotypes and highlight remodeling of protein assemblies involved in chromatin regulation in basal BC (Figs. 1c, 2a and 3a; Table 1 and Supplementary Tables 3 and 4). Status and activity of these complexes represents an important aspect in studies that aim to assess efficacy of epigenetic-targeted therapies. Patient stratifications built around molecular signatures are instrumental for guiding effective treatment decisions and new classes of signatures which encompass high-level biological information, such as protein complex status, could be of a special value for this. Moreover, the increasing capability to generate highly reproducible and quantitatively precise datasets with bottom-up proteomic methods will further benefit from new concepts for proteomics data analysis^4,30,31^.

## Methods

### Altered protein quantitative relationships

The AlteredPQR algorithm relies on the availability of a reference set, which is used to estimate a background distribution of values and a test set, which is assessed to find outliers from this distribution. The input file for the algorithm is a data matrix with log-transformed protein quantitative measurements (rows are protein identifiers and columns represent studied samples). Columns in the matrix which correspond to reference and test samples need to be defined by the user (see R package Vignette for examples). In addition, the algorithm relies on a list of protein pairs that are known to form stable interactions, such as those within a protein complex. The list should be based on previous knowledge or on inferred complex assignments. The one we used in this study is also available with the package, but the list can be replaced by the user. It is composed of human protein pairs that belong to the same CORUM protein complex^13^, are assigned as direct or indirect complex in Reactome Database^12^, are predicted to form a stable complex in Interactome3D database^14^ or are reported to stably interact in two or more affinity-purification MS studies^15^. For each of these pairs, a log ratio of their protein quantities was calculated for all studied samples (protein quantities were subtracted when the log-transformed matrix was used). Following, for each protein pair, the log ratio values in the reference samples were used to estimate a background distribution that captures technical and biological variability, and log ratio of protein quantities in each test samples was assessed to identify outliers from the background distribution. This was performed using the modified *z* score statistic:

$${Mi}=\frac{0.6745({x}_{i}-\widetilde{x})}{{MAD}}$$

Where:

*Mi* indicates a modified z score

χ_i_ indicates a log ratio of protein quantities for each protein pair in an individual test sample

$\widetilde{x}$ represents the median of the log ratio values for the same protein pair in the reference samples.

Protein pairs with an absolute (Mi) value >3.5 in individual test samples were considered as significant outliers. This threshold was proposed by Iglewicz and Hoaglin, based on their simulation study in which they calculated a proportion of random pseudo-normal observations classified as outliers based on 10,000 replications, in sets of the sample sizes 10, 20 and 30 (ref. ^16^). The threshold 3.5 is widely accepted in different implementations of the test. Of note, this threshold can still be associated with >5% of false-positive outlier values depending on the sample size. AlteredPQR scores were obtained by summing up significant outlier values. To associate the scores to *P* values, a background sample set composed of randomly sampled reference values was generated, analyzed proteins were grouped into random pairs, and AlteredPQR scores were calculated for these datasets. Distribution of the background scores was estimated with descdist and fitdist functions from the fitdistrplus R package (v.fitdistrplus_1.1-3). As this reported gamma distribution features, *P* values for the AlteredPQR scores obtained for the real datasets were calculated with the pgamma function using the estimated shape and rate of the simulated background distributions obtained by the fitdist function implementation of the matching moments method. The *P* values were adjusted for multiple testing with the BH method (Supplementary Fig. 4). For the analyses described in the manuscript text, threshold of adjusted *P* < 0.1 was used. In both BC cell line and patient samples analysis, this corresponded to about top 1% of AlteredPQR scores for the analyzed protein pairs. We also recommend considering protein pairs within the top 1% of the scores as likely biologically relevant candidates. For further discussion on the interpretation of results see Supplementary Note 4.

This list of outlier pairs includes also instances where one protein was strongly up- or downregulated and consequentially all protein pairs that include this protein were detected as significant. To exclude instances where multiple protein pairs were detected due to up- or downregulation of a single protein and compose a non-redundant list of protein pair outliers, we assessed the contribution of each protein to the signal. Specifically, for all test samples in which PQRs for the analyzed protein pair were detected as outliers, we tested whether in the same samples individual proteins had shifted values compared to their expression levels in the reference samples. The criteria to consider that a protein was contributing to the change in log ratio values for a protein pair was the following: at least half of the samples detected as outliers for the protein pair also had to be detected as outliers for a single protein, but with a lower threshold (we did not want to exclude pairs where both proteins had a mild, but opposite quantitative change which contributed to the outlier signal). Here, for individual proteins, we required an absolute modified *z* score for log protein values in the respective test samples to be higher than 2, when compared to the protein log quantities in reference samples. Each protein that passed this requirement was represented with a protein pair that had an overall highest sum of significant absolute *z* scores. By choosing representative protein pairs, it was avoided that the resulting list is dominated with instances where a single protein with many known interaction partners is over or under expressed.

In addition, proteins and protein pairs which were highly variable in BC cell line luminal samples, were excluded from the outliers list. Filtering for variability was used to overcome the limitation of a small reference set size and avoid noise. Criteria for this were:

(1) pairs which could be classified as outliers in three or more luminal samples according to the same thresholds used for non-luminal samples; that is the absolute modified *z* scores higher than 3.5 and (2) pairs that included proteins which in at least three luminal samples had values higher or lower than the median of log protein quantity in luminal samples ±1.4826 × 2-times its MAD in luminal samples.

Furthermore, for the following analyses, proteins associated with cytoskeleton functions were excluded to avoid instances where changes in protein expression reflect variable contribution of tumor stroma. These encompassed proteins annotated with the GO term GO:0005200. In addition, ribosomal proteins (those with the term ‘ribosomal’ in the protein description) were also excluded, due to the lack of isoform-specific annotations and hence inability to annotate the functional impact of the altered PQRs. This included all proteins annotated with the GO term GO:0003735.

For protein pairs detected as outliers, Pearson correlation of expression levels was calculated separately in reference samples and separately in all tested samples, as well as only in tested samples with altered PQRs.

Values in reference luminal samples were tested for normal distribution with the Shapiro–Wilk test. Overall, 92% and 84% of protein pairs in the analyzed BC cell line and patient samples, respectively, had a *P* value higher than 0.05, implying that the distribution of the data was not significantly different from normal distribution. Modified *z* scores require approximately normal distribution of values.

### Detection of functionally enriched categories

The AlteredPQR algorithm was applied on several quantitative proteomics datasets. To identify cellular functions which were most affected by the AlteredPQRs, proteins in the resulting lists were annotated with their KEGG and Reactome pathway assignments. To identify significantly enriched terms background sets were composed of proteins which were significant on the individual level, and all other measured proteins that entered the analysis, that is proteins that were listed as members of protein complexes. The former set of individually significant proteins was composed by performing Wilcoxon test between reference and test datasets and by considering only measured proteins that were also members of protein complexes. Enrichment of KEGG terms between proteins with altered PQRs and those in background sets was assessed using two-sided Fisher’s exact test. The obtained *P* values were corrected for multiple testing with the BH method. Enriched Reactome terms were obtained by uploading significant hits and background of analyzed proteins in the ConsensusPathDB database. There, significant *P* values are calculated with the hypergeometric test and corrected for multiple testing with FDR^32^.

Differences among the three sets with respect to the fraction of known cancer drivers (obtained from the Cancer Gene Census list^33^), BC-associated genes (obtained from DisGeNET annotations^34^ or Helios software assignments^35^) and BC subtype-specific essential genes (reported elsewhere^36^) were assessed with the chi-squared test.

### Drug response differences

To assess differences in drug responses, area under the curve (AUC) values, which corresponded to BC cell survival and proliferation after drug treatment, were obtained from a published study^9^. Basal cell lines which were used as a test set were divided in those with perturbed ratio and all others. AUC values were then compared between the two groups of cell lines for every protein pair–drug combination using the Wilcoxon test. The obtained *P* values were corrected with the BH method.

### Mapping of overrepresented protein complexes

To identify protein complexes with multiple subunits affected by altered PQRs, proteins with significant PQR changes were grouped according to their CORUM assignments^13^. Next, using a two-sided Fisher’s exact test, it was assessed whether any of the protein complexes were overrepresented on the resulting list.

In addition, it was assessed whether the whole protein complexes were also up- or downregulated in the tested samples. For this, only CORUM complex subunits measured across the majority of samples were considered and only samples in which all of the selected subunits were measured were considered further. Median expression levels of these subunits were used to represent protein complex expression. A procedure analogous to the one described above was used to identify outliers: median and MAD of protein complex expression levels in the reference samples were calculated and protein complex expression values in the individual test samples were compared to these values to identify significant outliers. Only complexes whose expression levels were categorized as outliers in 10% or more of the tested samples were considered further.

### Inference of differentially active cellular pathways

Pathway activity scores in samples from patients with BC for which quantitative proteomics data were analyzed were obtained from the same study^18^. For each protein pair with a significantly altered PQR, all studied samples were divided into two categories according to the quantitative ratio of the two proteins, which is based on whether this value was considered as an outlier in the respective sample or not. Pathway activities in the two categories were compared for all pathways with the assigned scores and, when any pathways with differences in their activities were found, the three most-significant pathways per pair were listed. To avoid redundancy, pathways whose proteins overlapped (>80%) with more significant pathways were omitted.

### Breast cancer subtype-specific PQR alterations

Protein pairs with significantly altered PQRs between the patient samples with luminal A subtype and all other samples were further assessed to identify whether any of these were subtype specific. Their quantitative ratios were compared between the subtypes using a Wilcoxon test. Samples in which the tested pairs had most significant differences in PQRs between the luminal B and basal samples were clustered and visualized using the R heat map tool.

### Annotation of proteins involved in epigenetic complexes

It was considered that proteins have a role in epigenetic regulation if they were cataloged as ‘Chromatin modifying enzymes’ in Reactome or annotated either with a GO molecular function term ‘Chromatin binding’ or a GO biological process term ‘Chromatin remodeling’ (Table 1).

### Reporting summary

Further information on research design is available in the Nature Portfolio Reporting Summary linked to this article.

## Online content

Any methods, additional references, Nature Portfolio reporting summaries, source data, extended data, supplementary information, acknowledgements, peer review information; details of author contributions and competing interests; and statements of data and code availability are available at 10.1038/s41592-023-02011-w.

### Supplementary information

Supplementary Information Supplementary Notes 1–4, Figs. 1–4, and legends of supplementary tables and description of supplementary software.

Reporting Summary

Supplementary Software R package for the inference of protein complex states from quantitative proteomics data. The package takes information on known stable protein interactions (that is protein components of the same complex) and assesses how protein quantitative ratios change between different conditions. It reports protein pairs for which relative protein quantities to each other have been significantly altered in the tested condition.

Supplementary Tables Supplementary Tables 1–4.

## Data Availability

Datasets analyzed here are available as supplementary information in previously published studies^9,18^ (available as a Supplementary Table 2 in Lapek et al.^9^ and Supplementary Table 3 in Mertins et al.^18^). A smaller example dataset for the test analysis is available within the R package. All other data described here are available upon a reasonable request. To perform statistical analyses we used Cancer Gene Census database (https://cancer.sanger.ac.uk/census), KEGG Pathway database (https://www.genome.jp/kegg/pathway.html), Reactome database (https://reactome.org), DisGeNET database (https://www.disgenet.org), BC driver predictions with the available as supplementary information in Sanchez-Garcia et al.^36^ (https://pubmed.ncbi.nlm.nih.gov/25433701/), GO annotations (http://geneontology.org), BC cell line survival data from Lapek et al.^9^ and patient pathway activities from Mertins et al.^18^, protein complex annotations from the CORUM (http://mips.helmholtz-muenchen.de/corum), Reactome (https://reactome.org) and Interactome3D (https://interactome3d.irbbarcelona.org) databases as well as stable interactions reported by multiple studies from the BioGRID database (https://thebiogrid.org).

## Code availability

Code is available as an R package AlteredPQR and stored at the CRAN repository at https://cran.r-project.org/web/packages/AlteredPQR/index.html.
