# Supplementary material for: A computational framework for the inference of protein complex remodeling from whole-proteome measurements
Source: Nat Methods. 2023 Sep 25;20(10):1523–9. doi: 10.1038/s41592-023-02011-w (PMC10555833; doi:10.1038/s41592-023-02011-w)
Supplement: Supplementary file 1 — Supplementary Notes 1–4, Figs. 1–4, and legends of supplementary tables and description of supplementary software. [file 41592_2023_2011_MOESM1_ESM.pdf]

nature methods

Article https://doi.org/10.1038/s41592-023-02011-w

A computational framework for the inference of protein complex remodeling from whole-proteome measurements

In the format provided by the authors and unedited

Supplementary Figures, Software, Tables and Notes

SUPPLEMENTARY FIGURES

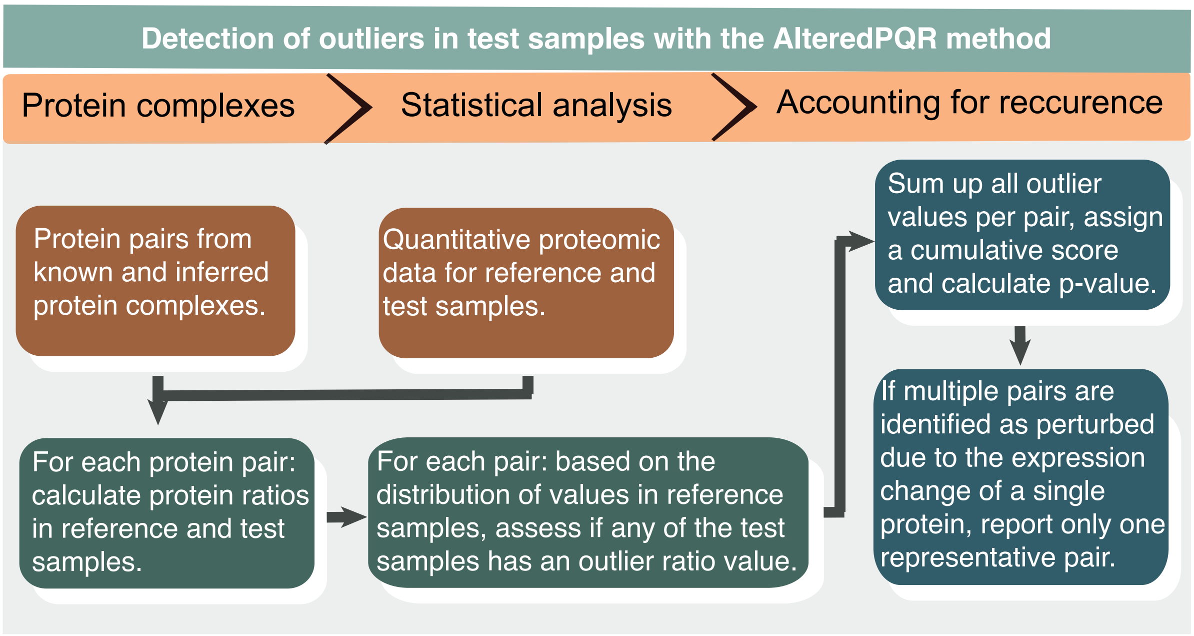

Supplementary Figure 1: Outline of the workflow to detect altered regulation in protein quantitative relationships (PQRs). The outline of individual steps in the AlteredPQR approach is presented. First, MS proteomic measurements are obtained and a list of protein pairs that are likely to interact is composed. For each interacting pair, protein quantitative relationship is summarized as a ratio of their expression values (or correspondingly, as a difference of log-scaled values). Distribution of the ratio values in the *reference samples* is represented with Median and MAD statistics. Outliers in the *analyzed set of test samples* are estimated by calculating modified z-scores for the quantitative ratios of the same pairs in each test sample. Finally, protein pairs are ranked based on the sum of z-scores across all significant samples. This gives more significance to perturbations found in multiple analyzed samples. In order to avoid reporting multiple protein pairs for single up- or down-regulated proteins, only one representative pair with the most significant score is listed for each protein which is driving the altered PQR signal.

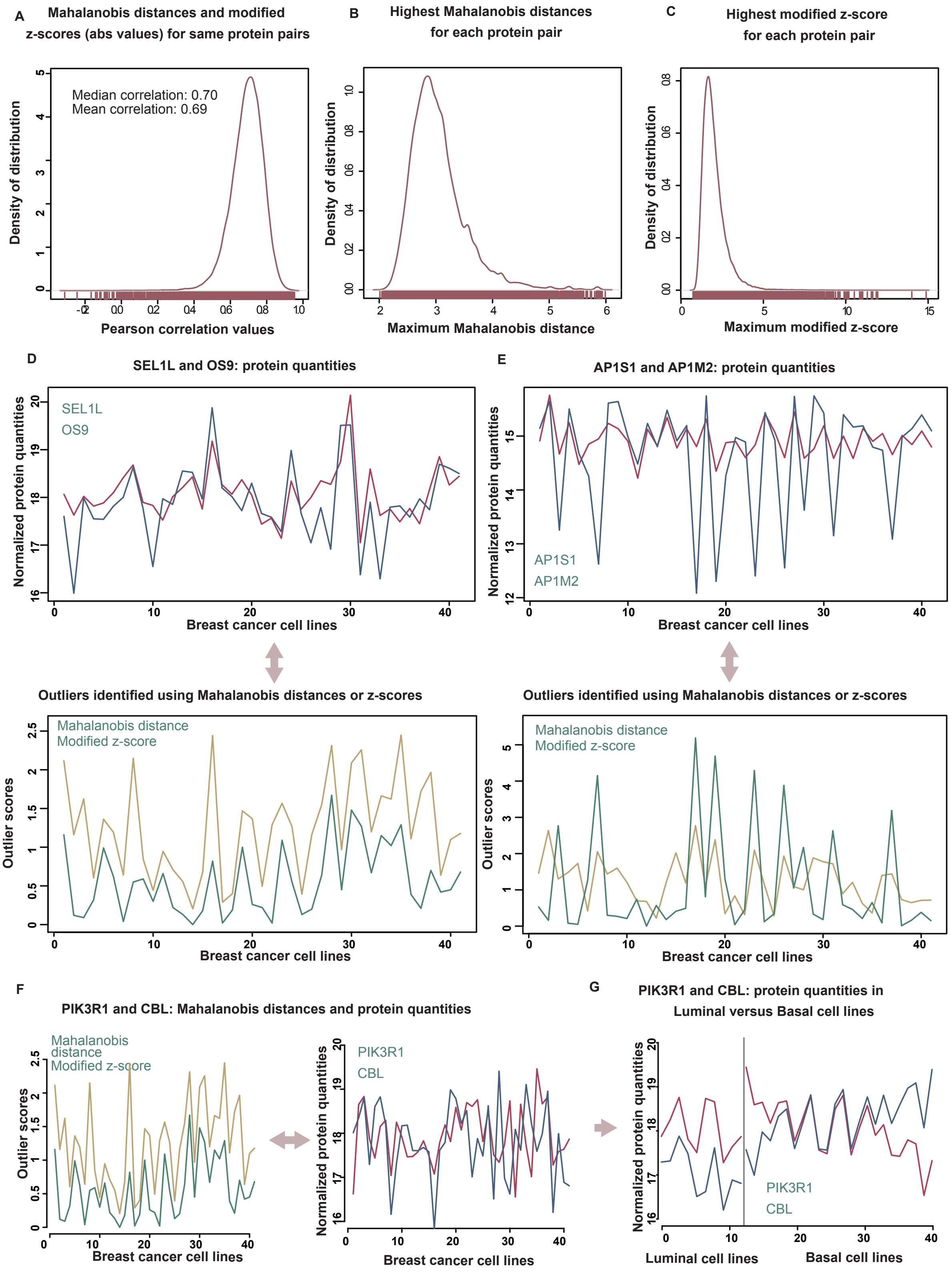

A
Mahalanobis distances and modified z-scores (abs values) for same protein pairs
Median correlation: 0.70
Mean correlation: 0.69
Density of distribution
Pearson correlation values
B
Highest Mahalanobis distances for each protein pair
Density of distribution
Maximum Mahalanobis distance
C
Highest modified z-score for each protein pair
Density of distribution
Maximum modified z-score
D
SEL1L and OS9: protein quantities
SEL1L
OS9
Normalized protein quantities
Breast cancer cell lines
E
AP1S1 and AP1M2: protein quantities
AP1S1
AP1M2
Normalized protein quantities
Breast cancer cell lines
Outliers identified using Mahalanobis distances or z-scores
Mahalanobis distance
Modified z-score
Outlier scores
Breast cancer cell lines
Outliers identified using Mahalanobis distances or z-scores
Mahalanobis distance
Modified z-score
Outlier scores
Breast cancer cell lines
F
PIK3R1 and CBL: Mahalanobis distances and protein quantities
Mahalanobis distance
Modified z-score
Outlier scores
Breast cancer cell lines
PIK3R1
CBL
Normalized protein quantities
Breast cancer cell lines
G
PIK3R1 and CBL: protein quantities in Luminal versus Basal cell lines
Normalized protein quantities
PIK3R1
CBL
Luminal cell lines
Basal cell lines

Supplementary Figure 2: Comparison of outliers identified using either Mahalanobis distances or modified z-scores and the reference-based approach.

(A) For the same protein pairs two outlier measures were used and compared: Mahalanobis distances and modified z-scores. To make them directly comparable, in this panel both scores were calculated using the reference-free approach, i.e. using the same population of samples – in this case BC cell lines – both as a reference and test set. As expected, the two outlier scores (Mahalanobis score and the absolute value of the modified z-score) correlate well across the protein pairs (the mean Pearson correlation score across all measured pairs was 0.69). Here, we analyzed only protein pairs that can be found within the same protein complex (criteria described in the main text).

(B) Distribution of maximum Mahalanobis scores for each tested pair protein is shown. High Mahalanobis scores indicate outlier cell lines. Distribution of maximum scores shows that with lower thresholds, the majority of tested samples will have at least one cell line detected as an outlier.

(C) Distribution of maximum values of the absolute modified z-score for each analyzed protein pair. The number of outliers also depends on the z-score threshold. For instance, only a minority of the analyzed pairs has at least one z-score that is higher than five.

(D) An example of a protein pair for which there was a disagreement between the two methods in the estimate of a cell line that was the strongest outlier, but where Mahalanobis and modified z-scores correlated well overall. Graphs above in the panel show expression levels of two proteins in the pair and the graphs below the associated Mahalanobis and z-score outlier scores. Overall, both scores were able to capture significant changes in expression patterns, with Mahalanobis being able to better highlight few significant changes, but also overestimating some differences.

(E) An example of a protein pair for which there was a disagreement in the estimate of a cell line with the maximum outlier score, and an overall low correlation between the Mahalanobis and modified z-scores (AP1S1 and AP1M2). Graphs above in the panel show expression levels of the two proteins in the pair and graphs below the associated Mahalanobis and outlier scores. Overall, both scores were able to capture significant changes in expression patterns and the modified z-score highlighted few significant changes more strongly.

(F) Mahalanobis and modified z-scores for the PIK3R1 and CBL protein pair calculated with a reference-free approach, i.e. all analyzed BC cell lines are used to obtain the reference statistics and identify outliers. On the right, expression levels of the two proteins are shown. Neither modified z-scores nor Mahalonobis distance outlier scores are standing out as significantly high.

(G) The PIK3R1 and CBL protein pair is among the most significant hits in the modified z-score analysis when Luminal BC cell lines are used to calculate the reference median and median absolute deviation (reference-based approach) and when basal cell lines are used as a test set to identify outliers. When the expression levels of the proteins are separated per Luminal and Basal cell lines, it is clear that in a subset of Basal cell lines, the two proteins have a very different expression pattern than in Luminal cells, and that CBL becomes more highly expressed than PIK3R1. Of note, in Supplementary Table 2 we show usage of Mahalanobis scores in the reference-based approach. For this, the population mean and covariance matrix calculated for the BC luminal reference set was used in the the Mahalanobis equation and outliers were searched for in the test set of Basal BC.

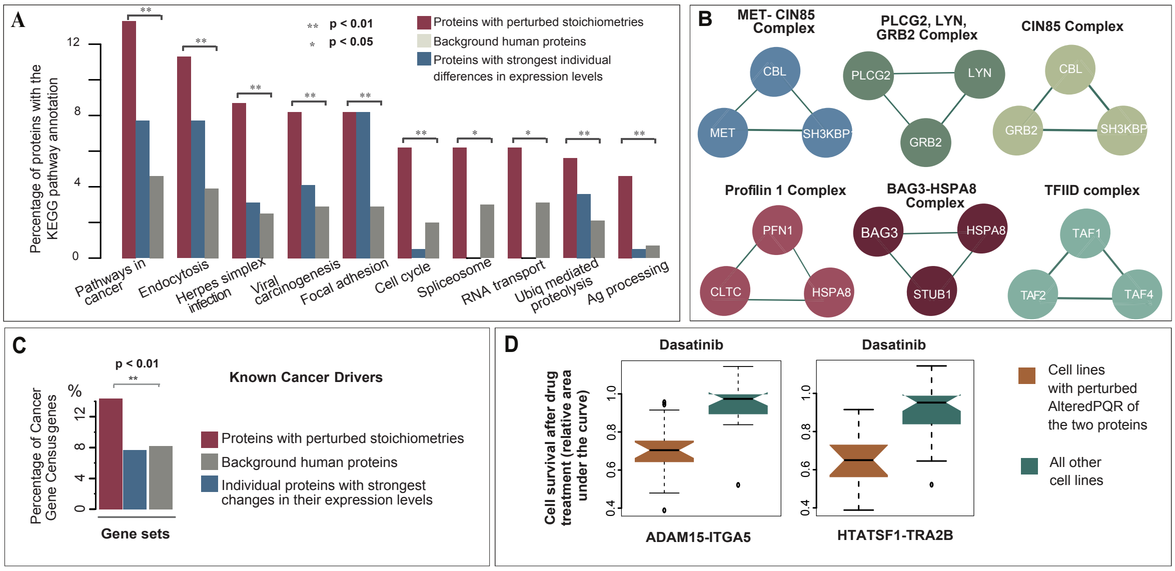

Supplementary Figure 3: Validation of the new approaches for outlier detection on the whole-cell lysate proteomics measurements for BC cell lines.

(A) KEGG pathway annotations, which are strongly enriched among the proteins that had altered PQRs detected by the reference-based approach (i.e. outliers in basal cell lines compared to reference values in luminal cell lines) are shown (dark red). These proteins are compared to (i) proteins that individually had highly significant expression changes between the two types of BC cell lines (dark blue), and (ii) to all other human proteins (grey). For an objective comparison, the two latter sets included only proteins that entered the **Altered_PQR** analysis. The indicated adjusted p-values represent a comparison with all proteins that entered the analysis (* indicates adjusted p < 0.05 and ** p < 0.01). P-values were calculated with the two-sided Fisher's exact test and adjusted with the Benjamini-Hochberg method. Adjusted p-values for the KEGG terms Pathways in cancer, Endocytosis, Herpes simplex infection, Viral carcinogenesis, Focal adhesion, Cell cycle, Spliceosome, RNA transport, Ubiquitin mediated proteolysis and Antigen processing and presentation are 3.09 $x10^{-5}$, 8.62 $x10^{-5}$, 8.62 $x10^{-5}$, 0.0006, 0.0006, 0.0014, 0.0341, 0.0341, 0.0061 and 8.62 $x10^{-5}$, respectively,

(B) Protein complexes overrepresented among the proteins with altered PQRs are shown. Overrepresented protein complexes were identified by comparing the fraction of subunits of each complex among the perturbed protein pairs to the fraction of complex subunits in the background set of all analyzed proteins. P-values were calculated with the Fisher's exact test and adjusted with BH method. All shown complexes were detected with an adjusted p-value < 0.02.

(C) Proteins with altered PQRs are also enriched in known cancer drivers (i.e. proteins included in the Cancer Gene Census database) when compared to proteins that individually had a strong expression change or all other proteins included in the analysis. The shown p-value (p < 0.00196) for a comparison to the background set of human proteins was calculated with a two-sided Pearson's Chi-square test.

(**D**) For each significant protein pair, area under the curve (AUC; indicating the cell number or cell survival) in cell lines in which the pair had an altered PQR was compared to AUC in other cell lines after the treatment with different drugs (average of two biological replicate measurements is shown). Dasatinib, a drug that was previously evaluated for the treatment of aggressive breast cancer elicited significantly different response in BC cell lines with perturbed PQRs compared to all other BC cell lines as illustrated with the boxplots here. P-values for the survival difference were obtained with the two-sided Mann-Whitney test and corrected for multiple testing by BH method. For the ADAM15 and ITGA5 protein pair, there were 19 (dark red boxplots) and 22 (green boxplots) biologically different BC cell lines with altered and non-altered PQR, respectively, which were treated independently with Dasatinib. Median AUC, which indicated cell survival, after drug treatment was 0.70 in the BC cell lines with the ADAM15-ITGA5 altered PQR and 0.97 in other BC cell

lines (adjusted p-value < 0.017). The minimum values for the two groups were 0.4 and 0.5, the boxes are bound by the lower quartiles of 0.6 and 0.9 and by the upper quartiles of 0.8 and 1.0, and their maximum values were 1.0 and 1.1). In addition, basal cell lines with altered PQR for the ADAM15-ITGA5 pair differed in the drug response when compared to other basal cell lines (p < 0.006, two-sided Mann-Whitney test, no multiple test correction, 19 basal cell lines with altered PQR and 4 without).

For the HTATSF1 and TRA2B protein pair, there were 12 (dark red boxplots) and 29 (green boxplots) biologically different BC cell lines with altered and non-altered PQR, respectively, which were treated independently with Dasatinib. Median AUC after drug treatment was 0.70 in the BC cell lines with the HTATSF1-TRA2B altered PQR and 1.0 in other BC cell lines (adjusted p-value < 0.025). The minimum values for the two groups were 0.4 and 0.5, the boxes are bound by the lower quartiles of 0.6 and 0.8 and by the upper quartiles of 0.7 and 1.0, and their maximum values were 0.9 and 1.1). In all boxplots, lower and upper whiskers are calculated by the formula: max(min(x), lower quartile – 1.5 * interquartile range) and min (max(values), upper quartile + 1.5 * interquartile range), respectively. In addition, basal cell lines with altered PQR for the HTATSF1-TRA2B pair differed in the drug response when compared to other basal cell lines (p < 0.005, two-sided Mann-Whitney test, no multiple test correction, 12 basal cell lines with altered PQR and 11 without).

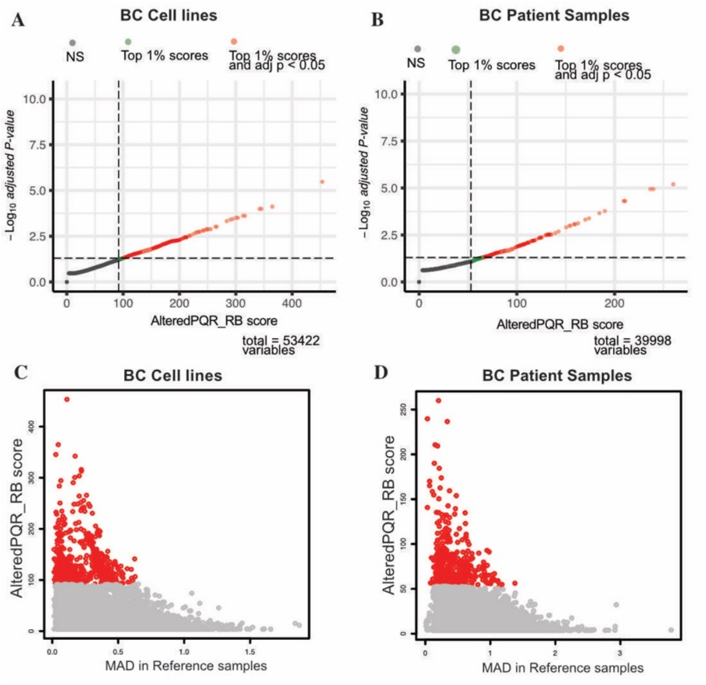

Supplementary Figure 4: Distribution of RB AlteredPQR scores and adjusted p-values and relationship between Median Absolute Deviation (MAD) in reference samples and AlteredPQR_RB score in tested samples. A and B: Based on the distribution of AlteredPQR values for the stimulated background sets calculated on randomly sampled reference Luminal cell lines or reference Luminal A patient samples, p values for the values obtained for the real datasets were calculated based on the estimated gamma distribution of background samples. P-values were adjusted with the BH method. Red dots represent Altered PQR scores where adjusted $p < 0.05$, green dots top 1% of all scores which corresponds to $0.05 < p < 0.1$ and grey all other scores (i.e. AlteredPQR scores for 99% of the analysed protein pairs). The majority of AlteredPQR scores are zero or close to zero. **C and D:** Highly significant AlteredPQR scores (adjusted $p < 0.05$) are colored in red. Protein pairs with a high variation in reference samples (i.e. high MAD), that is underlined either with technical properties or biological noise, are less likely to be associated with high AlteredPQR scores. A and C panels show BC cell lines and B and D show patient samples. P-values associated with the AlteredPQR scores were calculated in a comparison to a background gamma distribution using the R function ***pgamma.*** The p-values were adjusted for multiple testing with the BH method.

SUPPLEMENTARY SOFTWARE

Supplementary Software AlteredPQR. R package for the inference of protein complex states from quantitative proteomics data. The package takes information on known stable protein interactions (i.e. protein components of the same complex) and assesses how protein quantitative ratios change between different conditions. It reports protein pairs for which relative protein quantities to each other have been significantly altered in the tested condition.

SUPPLEMENTARY TABLES

Supplementary Table 1: Classification and disease prognosis for patients with breast cancer. Molecular markers and prognosis for the breast cancer subtypes discussed in the text are shown. However, immunohistochemistry markers are only surrogate markers for the molecular subtypes. The latter are better defined by large gene expression profiles (Perou et al. Nature 2000; the Cancer Genome Network Atlas Nature 2012) and there is never a 100% overlap.

Supplementary Table 2: Altered PQRs in the collection of breast cancer cell lines characterized with the MS-based proteomics.
(A) Comparison of top 1% protein pairs (534/53422 tested pairs) detected as outliers with the AlteredPQR method and reference-based implementation of the Mahalanobis method reveals a strong overlap. Protein pairs with the highest AlteredPQR score or the highest sum of significant Mahalanobis scores (threshold for significant was defined as top 1 quantile of all calculated values, or top 10 quantile of all values). Mahalanobis scores were calculated on non-Luminal BC cell lines using Luminal BC cell lines (classified as ERBB-) as a reference set.
(B) Protein pairs with altered PQRs in basal BC cell lines identified by using values in luminal BC cell lines to obtain a reference distribution. Significant protein pairs are listed together with information on the individual ranking of the analyzed proteins in a comparison of protein expression levels between luminal and basal cell lines. If the identified proteins are annotated as known cancer drivers (i.e. they are in the Cancer Gene Census), this information is included in the table. P-values are obtained based on a comparison to the distribution of scores calculated for the randomly sampled reference values for the analyzed proteins using gamma distribution test. They are adjusted for multiple testing by the Benjamini Hochberg method.
(C) Difference in the response to drugs between cell lines with altered PQRs and all other cell lines. Most significant protein pair and drug combinations are listed. Difference in the area under the curve (AUC) values represents a difference in cell numbers between cell lines that had a significantly altered PQR and other cell lines after a treatment with a drug. Adjusted p-values indicate a statistical difference in AUC calculated by the two-sided Mann-Whitney test and corrected for multiple testing of all analyzed protein pair - drug combinations.

Supplementary Table 3: Altered PQRs in the patients with more aggressive forms of breast cancer.
(A) Protein pairs with altered PQRs in the analysis of BC patient samples. Luminal A samples are used for obtaining a refrence distribution of values for the RB analysis, and all other subtypes are analyzed to look for the outliers from these values. Information on the individual ranking of the analyzed proteins in a comparison of protein expression levels between Luminal A and all other samples (collective comparison), or between Luminal A and different individual subtypes is also included. If the identified proteins are annotated as known cancer drivers (i.e. they are in the Cancer Gene Census), this information is also included in the table. P-values are obtained based on a comparison to the distribution of scores calculated for the randomly sampled reference values for the analyzed proteins using gamma distribution test. They are adjusted for multiple testing by the Benjamini Hochberg method.

(B) CORUM protein complexes in which the majority of subunits was strongly up- or down-regulated in non-Luminal A BC patient samples. Protein complex expression levels were estimated as the median expression levels of their measured subunits (only subunits measured across multiple samples, which hence made the complex levels comparable across samples, were considered). Protein complexes whose expression levels in more aggressive forms of BC were outliers compared to LumA samples were identified using the modified z-score statistics and the outlier thresholds for modified z-scores in individual samples of >3 and <-3.
(C) Pathways with the highest activity score differences between the samples with altered PQRs and all other samples. Top 10 pathways for every pair with an altered PQR are shown. P-values are calculated with the two-sided Mann-Whitney test and corrected for multiple testing with the BH method. Protein members of pathways significant for the same pair often strongly overlap.

Supplementary Table 4: Protein pairs that can form stable interactions and correlate strongly in basal, but not in luminal A patient samples, or the other way around. Pearson correlation was calculated for all pairs with sufficient measurements in basal and luminal A samples. Pairs that correlated significantly either in luminalA or in basal samples (Perason correlation higher than 0.6 and correlation p-value < 0.01) and that showed a significant shift in correlation values (>0.6) between luminal A and basal samples are shown. The shown p-values were calculated with a two-sided correlation test implemented in the R function cor.test, using Pearson's coefficients. They were adjusted for multiple testing with the BH method.

SUPPLEMENTARY NOTES

Supplementary Note 1
Validation of the method for detecting PQR alterations on MS proteomic data of a breast cancer cell line compendium

To assess the performance of the method, we used a publicly available proteomic dataset of 41 BC cell lines composed of 24 basal and 17 luminal cell lines[1] with abundance data for more than 6,900 proteins[1]. We used measurements in luminal (i.e. hormone receptor positive) BC cell lines as a reference set (we considered only 10 luminal cell lines which were also ERBB negative) and looked for significant outliers among the basal BC cell lines by applying the AlteredPQR algorithm. Significant scores (absolute (Mi) > 3.5) were summed up and p-values calculated through a comparison to the same size background reference set composed of randomly sampled protein quantities in luminal samples (see Methods). Benjamini-Hochberg (BH) adjusted p-values <0.1 corresponded to about 1% of all analyzed pairs. We excluded instances where multiple pairs are reported due to dysregulation of a single protein and filtered out less-specific GO terms related to ribosomal or cytoskeleton function (see Methods). In total, 116 protein pairs with 195 proteins had strongly altered PQRs in different subsets of basal cell lines (**Supplementary Table 2**). Changes in PQRs represent a novel concept compared to standard analysis of individual proteins with up- or down-regulated expression levels. Therefore, we also compared proteins with altered PQR in basal cell lines to the same size set of proteins that were individually strongly differentially expressed between the basal and luminal cells, and to the background set of all proteins that entered the analysis (see Methods). Only 25% (i.e. 48) of proteins with altered PQRs were among the 195 topmost differentially expressed proteins (**Supplementary Table 2**). Compared to the two other sets (after filtering for same protein classes to make comparisons objective), proteins with altered PQRs were highly enriched in cellular processes with cancer-associated roles, with the most significantly enriched KEGG terms including "Pathways in cancer" (**Supplementary Fig. 3A**, BH adjusted p-value < 3.1×10^{-5}, Fisher's exact test). We assessed if any specific protein complexes were overrepresented among protein pairs with altered PQRs using CORUM protein complex annotations (see Methods). This showed that by comparing basal to luminal cell lines, we could

identify changes in CIN85 complex that is important for cellular invasion and associated with aggressive BC phenotypes[2] (**Supplementary Figure 3B**), as well as in other assemblies that were suggested to have roles in breast cancer progression, such as Profilin 1 and PLCG2, LYN and GRB2 complexes (**Supplementary Figure 3B**)[3,4]. Proteins with altered PQRs also contained a high fraction of known cancer drivers (**Supplementary Figure 3C**, p-value < 0.004, when compared to the background set of other analyzed proteins, chi-square test). Overall, the benchmark application of the reference-based method showed that protein pairs identified as outliers in the BC basal cell lines were representative of biological characteristics that could distinguish them from luminal cells and that by analyzing the altered PQRs, it was possible to obtain information on the plausible remodeling of protein interactions, which was not readily evident from studying individual protein quantities.

In instances where the identified proteins do not play a causal role in disease progression, their perturbation could still serve as a biomarker for the given cellular state and possibly even predict a response to a stimulus or a drug treatment. For the BC cell lines studied here, there is data available on their sensitivity to different drugs[1]. We hence explored if the altered PQRs could serve as indicators of drug responsiveness. For each significant protein pair, we compared drug response between cell lines classified as outliers and all other cell lines. This highlighted as significant **43** protein pair-drug combinations (**Supplementary Table S6**, Wilcoxon test, BH adjusted p-value < 0.05). Altered PQRs of the protein pair HTATSF1-TRA2B were connected with a differential survival of basal BC cell lines after treatment with multiple different drugs. Previous studies have associated TRA2B expression levels with cancer cell survival and therapeutic sensitivity[5]. In addition, several drugs that were previously suggested for triple negative BC treatments showed opposite effects in basal cell lines in which the identified protein pairs were perturbed, compared to other basal cell lines (**Supplementary Table S6**). This included Dasatinib, a tyrosine kinase inhibitor, which is currently used as first and second line treatment of chronic myeloid leukemia[7] (**Supplementary Figure 3D)**. The drug has been proposed as a promising treatment for basal BC patients who do not respond to a chemotherapy[8]. However, the conducted clinical trial did not prove its efficacy on the unselected group of patients[9]. A possible application of PQR alterations could thus be in expanding the existing classes of biomarkers and guiding clinically relevant stratifications.

Supplementary Note 2
Application of *AlteredPQR* method on BC patient data: activity of cellular signaling pathways associated with the PQR status of related upstream proteins

Cultured cell lines often do not accurately represent clinical phenotypes and, over time, cell culturing can modify the molecular make up of cells[12]. Next, we investigated if the observed altered PQRs associated with molecular phenotype changes in the analyzed patient samples. For this, we used the phosphoproteomic dataset from the original study[13]. The data was integrated across signaling pathways and in this way, based on the phosphorylation states of proteins within a pathway, an activity score was assigned for more than 900 curated pathways in each patient sample[13]. For every protein pair identified as having an altered PQR in this study, we compared pathway activity scores between patient samples in which we detected the respective quantitative alteration to the pathway activity in all other samples (see Methods). The results highlighted several instances where changes in protein quantitative ratios affected a protein kinase and associated with altered signaling activity in functionally related cellular pathways (**Supplementary Table 3**). This is illustrated with the increased activities of Phosphoinositide (PI) 3 kinase and Vascular Endothelial Growth Factor (VEGF) pathways in samples in which PIK3CA and SRC have altered quantitative ratios[14] (BH adjusted p-value < 0.03, Wilcoxon test) and an increased activity of Cell cycle and DNA replication pathways in samples in which the quantity of Serine/Threonine Kinase

26 (STK26) is significantly increased over the Striatin 3 (STRN3) quantity (BH adjusted $p < 2x10^{-4}$, Wilcoxon test) (Figure 3E). SRC kinase is known to activate proteins in the PI and VEGF pathways and act synergistically with them in mediating progression of several cancer hallmarks[15,16]. Similarly, STK26 (also known as MST4) is a component of the STRIPAK complex and is known to be involved in cell growth and proliferation[17]. For the PIK3CA and SRC protein pair (adjusted p-value < 0.0011), there were 22 biologically independent patient samples (dark red boxplots in Figure 2C) with altered PQR and 58 samples (BC and normal breast tissue) without PQR alterations (green boxplots in Figure 2C). Median estimated activity scores for the PI pathway were -0.7 and 1.2 in the non-altered and altered groups, respectively (the minimum values for the two groups were -4.7 and -2.2, the boxes are bound by the lower quartiles of -1.8 and 0 and by the upper quartiles of 0.6 and 2.0, while the maximum values in the two groups were 3.0 and 2.9). Median estimated activity scores for the VEGF pathway (adjusted p-value < 0.0004) were -0.6 and 0.6 in the non-altered and altered groups, respectively (the minimum values for the two groups were -2.5 and -1.9, the boxes are bound by the lower quartiles of -1.1 and 0.1 and by the upper quartiles of 0.1 and 0.9, while the maximum values in the two groups were 1.5 and 2.6). For the STK26 and STRN3 protein pair, there were 22 biologically independent patient samples (dark red boxplots in Figure 2C) with altered PQR and 58 samples without PQR alterations (green boxplots). Median estimated activity scores for the cell cycle pathway (adjusted p-value < $4.96x10^{-5}$) were -0.9 and 1.5 in the non-altered and altered groups, respectively (the minimum values for the two groups were -5.6 and -1.7, the boxes are bound by the lower quartiles of -2.2 and 0.9 and by the upper quartiles of 0.2 and 3.1, while the maximum values were 3.8 and 6.0). Median estimated activity scores for the DNA replication pathway (adjusted p-value < 0.0002) were -0.9 and 0.9 in the non-altered and altered groups, respectively (the minimum values for the two groups were -5.5 and -1.4, the boxes are bound by the lower quartiles of -1.5 and 0.6 and by the upper quartiles of -0.1 and 1.7, while the maximum values were 4.0 and 3.8). In all boxplots, lower and upper whiskers are calculated by the formula: max(min(x), lower quartile – 1.5 * interquartile range) and min (max(values), upper quartile + 1.5 * interquartile range), respectively.

Of note, even though the AlteredPQR method does not explicitly check for correlations, the examples shown in the **Figure 1D** and **1E** had a higher Pearson correlation in samples with perturbed quantitative relationships, than in the reference Luminal A samples (-0.1 vs 0.5, -0.4 vs 0.5, 0.18 vs 0.7 and -0.1 vs 0.3 for ZBTB17-DNMT3A, DNMT1-SIRT1, PIK3CA-SRC and STK26-STRN3, respectively). These results indicate that protein complex changes inferred from the quantitative relationships among its subunits could relate to the activity of associated cellular pathways.

In the main analysis, luminal B, HER2 and basal samples were compared to the luminal A reference set. We further compared altered PQR patterns in basal samples to those in the luminal B subtype. This showed that a small fraction of the identified altered PQR pairs was able to effectively separate the two BC subtypes (**Figure 1F**). Among the identified pairs were proteins involved in DNA damage response (MLH1, MSH2, MSH3, MSH6, USP7, TP53BP1, SUPT16H, CETN2, EPC2 and MDC1), chromatin regulation (MEAF6, SMARCC2, SMARCB1, CBX8, EPC2, KMT2A) and cell cycle (TP53, USP39, KRT18, SMARCB1, SUPT5H). Together, these analyses show that reference-based method can infer disease-associated changes in protein complex states. Moreover, protein complexes affected by the AlteredPQRs suggest a link between the aggressive BC phenotypes and a remodeling of master epigenetic regulatory complexes.

Supplementary Note 3
Remodeling of epigenetic complexes in more aggressive cancer manifestations

In this study, with both PQR outlier and correlation shift approaches, we detected a strong remodeling of epigenetic complexes around the HDAC2 protein in basal BC. HDAC2 was previously associated with aggressive forms of BC and several HDAC inhibitors are in clinical trials. Even though they primarily show promise for the treatment of gliomas and glioblastomas[18], they have also been considered as options for combinatorial treatments in BC[19,20]. In addition, a previous study has shown that a joint activity of DNMT1 and DNMT3B epigenetic regulators, together with components of the nucleosome remodeling and deacetylase NuRD complex, drives epigenetic silencing of multiple tumor suppressors in colon cancer[21]. Moreover, a synergistic inactivation of DNMTs together with the NuRD's subunit CHD4 depletion was able to reactivate the silenced tumor suppressors[21]. Here, we found that DNMT1 and DNMT3A proteins had strongly altered PQRs in more aggressive BC samples (**Figure 2A**) and we detected a strong correlation shift in basal BC samples for in total 6 of NuRD1/2 complex subunits (**Table 1**). In particular, the NuRD's subunits HDAC2 and CHD4 had a number of epigenetic interaction partners with whom they showed coordinated expression patterns only in basal BC (**Table 1**). Furthermore, even 11 proteins from the **Table 1** can be found within the ALL-1 histone methyltransferase, a large complex involved in leukemia development[22] and some of the complexes, such as HDAC and SIN3 are also known to form larger complex assemblies together. Of note, expression level of HDAC2 was previously reported to have an inverse relationship with the expression levels of multiple tumor suppressors[21]. Overall, our results indicate that formation of several epigenetic complexes centered on HDAC2 and CHD4 proteins may be important for the progression of basal BC.

Supplementary Note 4
AlteredPQR package usage and interpretation of results

In addition to changes in expression levels and posttranslational modifications of crucial proteins, cellular decision-making also depends on relative quantitative relationships among the expressed proteins[23,24]. This is particularly pronounced for proteins involved in the same pathway or protein complex[24,25]. With the increased precision and reproducibility of MS-based proteomics measurements that has been achieved over the last few years, it is now possible to generate a reliable quantitative data matrix, which forms the basis for analyzing quantitative relationships of protein complex subunits across different samples[26-29]. With this study, we demonstrate that such data can be used for inferring the state of protein modules. Furthermore, we find that quantitative relationships between proteins, which map to the same assemblies, associate with the activity of the related cellular pathways (**Fig. 2C**) and drug sensitivity of cancer cell lines (**Supplementary Fig. 3D**), thus underpinning the biological relevance of the observed quantitative alterations. To automate analyses of PQRs, we developed an R package **AlteredPQR**. The package supports finding of outliers in a test set and requries the assignment of a reference set of samples. Because the approach provides new biochemically relevant information from datasets generated by conventional proteomic measurements, it promises to increase the confidence in the detection of biomarkers. Moreover, the identified changes in expression patterns of protein complex subunits provide valuable insights into the status of cellular assemblies.

The observed relative changes in protein expression levels reflect the overall abundance of a protein in the cell, which is estimated from the quantitative levels of its peptides. Changes in gene expression levels, protein translation, degradation and localization, as well as peptide post-translational modifications or presence of mutations that interfere with complex formation, can all influence the observed changes in protein expression levels. The outliers identified by the

approaches described here can point to cellular changes associated with test- or disease-specific samples underlined by independent molecular mechanisms.

The estimated distribution of variability from the reference population we introduce here increases sensitivity in outlier detection and the implemented non-parametric approach provides robustness in the signal detection, which allows for smaller sample sets. However, the overall assumption of the used statistical test is that the reference values should still resemble normal distribution. In order to satisfy the requirement for a symmetry in the distribution of values, we would not recommend to use reference sets with fewer than 7 samples[30,31]. In addition, we noted that none of the protein pairs with a very high AlteredPQR scores (ranked in the highest 1%) had a significant variation in reference samples (**Supplementary Fig. 5C and D**). While it is beneficial to exclude outliers underlined with high technical variability and biological noise, we recommend users to assess this pattern in each study.

In order to reduce the search space and possible false negative results, we focused here on analyzing protein pairs that are known to participate in the same protein complex. However, the AteredPQR approach can also be applied to studying quantitative relationships among other proteins whose expression levels are co-regulated at certain conditions. This could include proteins involved in the same biological pathway or functional process or proteins encoded by the genes that are regulated by the same transcription factor or subjected to the shared chromatin conformation changes, or other instances of expected co-regulation. A default list of protein pairs, based on human protein complexes, is provided with the package but it can be replaced with a user-defined list. Of note, there are also strong differences with respect to quantitative relationships among detectable subunits of different protein complexes. For instance, evolutionary conserved subunits involved in core cellular processes, such as transcription, translation or metabolism, tend to have a higher correlation than complexes with specific signaling roles that rely on short-lived physical assemblies.

In conclusion, the new PQR outlier method described here shows power to detect perturbations in cellular interactions that can indicate a status and activity of a protein complex and point to disease-associated alterations in these. The method is available as an R package.

References

1. Lapek, J.D., *et al.* Detection of dysregulated protein-association networks by high-throughput proteomics predicts cancer vulnerabilities. *Nature biotechnology* **35**, 983-989 (2017).
2. Nam, J.M., *et al.* CIN85, a Cbl-interacting protein, is a component of AMAP1-mediated breast cancer invasion machinery. *EMBO Journal* **26**, 647-656 (2007).
3. Janke, J., *et al.* Suppression of tumorigenicity in breast cancer cells by the microfilament protein profilin 1. *J Exp Med* **191**, 1675-1686 (2000).
4. Dutta, B., *et al.* A network-based, integrative study to identify core biological pathways that drive breast cancer clinical subtypes. *Br J Cancer* **106**, 1107-1116 (2012).
5. Liu, T., *et al.* TRA2A Promoted Paclitaxel Resistance and Tumor Progression in Triple-Negative Breast Cancers via Regulating Alternative Splicing. *Molecular cancer therapeutics* **16**, 1377-1388 (2017).
6. Fang, Y., Yu, H., Liang, X., Xu, J. & Cai, X. Chk1-induced CCNB1 overexpression promotes cell proliferation and tumor growth in human colorectal cancer. *Cancer Biol Ther* **15**, 1268-1279 (2014).
7. Kantarjian, H., *et al.* Dasatinib versus Imatinib in Newly Diagnosed Chronic-Phase Chronic Myeloid Leukemia. *New England Journal of Medicine* **362**, 2260-2270 (2010).
8. Tian, J., *et al.* Dasatinib sensitises triple negative breast cancer cells to chemotherapy by targeting breast cancer stem cells. *British journal of cancer* **119**, 1495-1507 (2018).
9. Finn, R.S., *et al.* Dasatinib as a single agent in triple-negative breast cancer: results of an open-label phase 2 study. *Clinical cancer research : an official journal of the American Association for Cancer Research* **17**, 6905-6913 (2011).
10. Sameni, M., *et al.* Cabozantinib (XL184) Inhibits Growth and Invasion of Preclinical TNBC Models. *Clin Cancer Res* **22**, 923-934 (2016).
11. Musumeci, F., Greco, C., Grossi, G., Molinari, A. & Schenone, S. Recent Studies on Ponatinib in Cancers Other Than Chronic Myeloid Leukemia. *Cancers (Basel)* **10**(2018).
12. Liu, Y., *et al.* Multi-omic measurements of heterogeneity in HeLa cells across laboratories. *Nature Biotechnology* **37**, 314-322 (2019).
13. Mertins, P., *et al.* Proteogenomics connects somatic mutations to signalling in breast cancer. *Nature* **534**, 55-62 (2016).
14. Weddell, J.C., Chen, S. & Imoukhuede, P.I. VEGFR1 promotes cell migration and proliferation through PLCγ and PI3K pathways. *NPJ systems biology and applications* **4**, 1-1 (2018).
15. Fruman, D.A., *et al.* The PI3K Pathway in Human Disease. *Cell* **170**, 605-635 (2017).
16. Eliceiri, B.P., *et al.* Selective requirement for Src kinases during VEGF-induced angiogenesis and vascular permeability. *Molecular cell* **4**, 915-924 (1999).
17. Shi, Z., Jiao, S. & Zhou, Z. STRIPAK complexes in cell signaling and cancer. *Oncogene* **35**, 4549-4557 (2016).
18. Lin, G.L., *et al.* Therapeutic strategies for diffuse midline glioma from high-throughput combination drug screening. *Science Translational Medicine* **11**(2019).
19. Ediriweera, M.K., Tennekoon, K.H. & Samarakoon, S.R. Emerging role of histone deacetylase inhibitors as anti-breast-cancer agents. Vol. 24 685-702 (Elsevier Ltd, 2019).
20. Zucchetti, B., Shimada, A.K., Katz, A. & Curigliano, G. The role of histone deacetylase inhibitors in metastatic breast cancer. Vol. 43 130-134 (Churchill Livingstone, 2019).
21. Cai, Y., *et al.* The NuRD complex cooperates with DNMTs to maintain silencing of key colorectal tumor suppressor genes. *Oncogene* **33**, 2157-2168 (2014).
22. Nakamura, T., *et al.* ALL-1 is a histone methyltransferase that assembles a supercomplex of proteins involved in transcriptional regulation. *Molecular cell* **10**, 1119-1128 (2002).
23. Romanov, N., *et al.* Disentangling Genetic and Environmental Effects on the Proteotypes of Individuals. *Cell* **177**, 1308-1318.e1310 (2019).
24. Krug, K., *et al.* A Curated Resource for Phosphosite-specific Signature Analysis. *Molecular & cellular proteomics : MCP* **18**, 576-593 (2019).
25. Papp, B., Pál, C. & Hurst, L.D. Dosage sensitivity and the evolution of gene families in yeast. *Nature* **424**, 194-197 (2003).

26. Williams, E.G., *et al.* Systems proteomics of liver mitochondria function. *Science (New York, N.Y.)* **352**, aad0189-aad0189 (2016).
27. Röst, H.L., *et al.* OpenSWATH enables automated, targeted analysis of data-independent acquisition MS data. *Nature biotechnology* **32**, 219-223 (2014).
28. Xuan, Y., *et al.* Standardization and harmonization of distributed multi-center proteotype analysis supporting precision medicine studies. *Nat Commun* **11**, 5248 (2020).
29. Collins, B.C., *et al.* Multi-laboratory assessment of reproducibility, qualitative and quantitative performance of SWATH-mass spectrometry. *Nat Commun* **8**, 291 (2017).
30. Iglewicz, B.a.H., David. How to Detect and Handle Outliers. *The ASQC Basic References in Quality Control: Statistical Techniques* **16**(1993).
31. D'Agostino, R. & Pearson, E.S. Tests for departure from normality. Empirical results for the distributions of b 2 and√ b. *Biometrika* **60**, 613-622 (1973).
