# Supplementary material for: A computational framework for the inference of protein complex remodeling from whole-proteome measurements
Source: Nat Methods. 2023 Sep 25;20(10):1523–9. doi: 10.1038/s41592-023-02011-w (PMC10555833; doi:10.1038/s41592-023-02011-w)
Supplement: Supplementary file 2 — Reporting Summary [file 41592_2023_2011_MOESM2_ESM.pdf]

nature research

Corresponding author(s): Marija Buljan and Ruedi Aebersold

Last updated by author(s): July 07, 2023

Reporting Summary

Nature Research wishes to improve the reproducibility of the work that we publish. This form provides structure for consistency and transparency in reporting. For further information on Nature Research policies, see our Editorial Policies and the Editorial Policy Checklist.

Statistics

For all statistical analyses, confirm that the following items are present in the figure legend, table legend, main text, or Methods section.

n/a	Confirmed	
☐	☒	The exact sample size (*n*) for each experimental group/condition, given as a discrete number and unit of measurement
☐	☒	A statement on whether measurements were taken from distinct samples or whether the same sample was measured repeatedly
☐	☒	The statistical test(s) used AND whether they are one- or two-sided<br>*Only common tests should be described solely by name; describe more complex techniques in the Methods section.*
☐	☒	A description of all covariates tested
☐	☒	A description of any assumptions or corrections, such as tests of normality and adjustment for multiple comparisons
☐	☒	A full description of the statistical parameters including central tendency (e.g. means) or other basic estimates (e.g. regression coefficient) AND variation (e.g. standard deviation) or associated estimates of uncertainty (e.g. confidence intervals)
☐	☒	For null hypothesis testing, the test statistic (e.g. F, t, r) with confidence intervals, effect sizes, degrees of freedom and P value noted<br>*Give P values as exact values whenever suitable.*
☒	☐	For Bayesian analysis, information on the choice of priors and Markov chain Monte Carlo settings
☐	☒	For hierarchical and complex designs, identification of the appropriate level for tests and full reporting of outcomes
☐	☒	Estimates of effect sizes (e.g. Cohen's d, Pearson's r), indicating how they were calculated

Our web collection on statistics for biologists contains articles on many of the points above.

Software and code

Policy information about availability of computer code

Data collection	We did not collect new data for the study.
Data analysis	Data was analyzed with the R software tool AlteredPQR, which has been developed specifically for this study and which is described in the manuscript. The R software tool is available from the CRAN repository: https://cran.r-project.org/web/packages/AlteredPQR/index.html<br>For the reported statistical analyses, we used in-house written perl scripts and R code.

For manuscripts utilizing custom algorithms or software that are central to the research but not yet described in published literature, software must be made available to editors and reviewers. We strongly encourage code deposition in a community repository (e.g. GitHub). See the Nature Research guidelines for submitting code & software for further information.

Data

Policy information about availability of data

All manuscripts must include a data availability statement. This statement should provide the following information, where applicable:

- Accession codes, unique identifiers, or web links for publicly available datasets
- A list of figures that have associated raw data
- A description of any restrictions on data availability

Datasets analyzed here are available as supplementary information in previously published studies (i.e. available as a Supplementary Table 2 in Lapek *et al*. PMID: 28892078 and a Supplementary Table 3 in Mertins *et al*. PMID: 27251275). A smaller example dataset for the test analysis is available within the R package. All other data described here is available upon a reasonable request. To perform statistical analyses we used Cancer Gene Census database (https://cancer.sanger.ac.uk/census), KEGG Pathway database (https://www.genome.jp/kegg/pathway.html), Reactome database (https://reactome.org), DisGeNET database (https://www.disgenet.org), BC driver predictions with the available as supplementary information in Sanchez-Garcia *et al*. (https://pubmed.ncbi.nlm.nih.gov/25433701/), Gene Ontology annotations (http://geneontology.org), BC cell line survival data from Lapek *et al.* (PMID: 28892078) and patient pathway activities from Mertins *et al*. (PMID: 27251275), Protein complex annotations from the CORUM (http://mips.helmholtz-muenchen.de/corum), Reactome (https://reactome.org) and Interactome3D (https://interactome3d.irbbarcelona.org) databases as well as stable interactions reported by multiple studies from the BioGRID database (https://thebiogrid.org).

Field-specific reporting

Please select the one below that is the best fit for your research. If you are not sure, read the appropriate sections before making your selection.

☒ Life sciences ☐ Behavioural & social sciences ☐ Ecological, evolutionary & environmental sciences

For a reference copy of the document with all sections, see nature.com/documents/nr-reporting-summary-flat.pdf

Life sciences study design

All studies must disclose on these points even when the disclosure is negative.

Sample size	Sample size corresponded to all available samples with high quality proteomic measurements: 41 breast cancer cell lines and 77 patient biopsy samples. The described statistical methods do not explicitly require a normal distribution of values (for instance, we implemented a modified z-score instead of the z-score).
Data exclusions	We excluded only samples that were also excluded from the main conclusion in the original study, such as for instance patient samples that showed high level of degradation in the proteomic measurements.
Replication	Data on the cell line response to drugs (Supplementary Figure 3) included two replicates. Average of the values was taken for the analyses.
Randomization	The only additional information related to the studied cell lines and patient samples was a breast cancer subtype. Grouping of samples by the subtype was done for some of the analyses and this is then described in detail in the text.
Blinding	We were aware of the sample annotations (breast cancer subtype), but the same procedure was performed for all studied samples.

Reporting for specific materials, systems and methods

We require information from authors about some types of materials, experimental systems and methods used in many studies. Here, indicate whether each material, system or method listed is relevant to your study. If you are not sure if a list item applies to your research, read the appropriate section before selecting a response.

Materials & experimental systems

n/a	Involved in the study	
☒	☐	Antibodies
☒	☐	Eukaryotic cell lines
☒	☐	Palaeontology and archaeology
☒	☐	Animals and other organisms
☒	☐	Human research participants
☒	☐	Clinical data
☒	☐	Dual use research of concern

Methods

n/a	Involved in the study	
☒	☐	ChIP-seq
☒	☐	Flow cytometry
☒	☐	MRI-based neuroimaging
