# Supplementary material for: A computational framework for the inference of protein complex remodeling from whole-proteome measurements
Source: Nat Methods. 2023 Sep 25;20(10):1523–9. doi: 10.1038/s41592-023-02011-w (PMC10555833; doi:10.1038/s41592-023-02011-w)
Supplement: Supplementary file 3 — R package for the inference of protein complex states from quantitative proteomics data. The package takes information on known stable protein interactions (that is protein components of the same complex) and assesses how protein quantitative ratios change between different conditions. It reports protein pairs for which relative protein quantities to each other have been significantly altered in the tested condition. [file 41592_2023_2011_MOESM3_ESM.gz › AlteredPQR/inst/doc/AlteredPQR.pdf]

Vignette for the AlteredPQR package

Marija Buljan

June 9, 2023

The package infers changes in protein complex states from quantitative proteomics data. It takes information on known stable protein interactions (i.e. protein subunits of the same complex) and assesses how their protein quantitative ratios change between different conditions. It reports protein pairs for which relative protein quantities to each other have been significantly altered in the tested condition when compared to their reference levels.

1 Example run.

Load the **AlteredPQR** R package before starting the analysis.

```
> library(AlteredPQR)
```

The package provides example datasets, which can be used to run the functions AlteredPQR_RB and () and CorShift().

```
> data("int_pairs", package = "AlteredPQR")
> data("quant_data_all", package = "AlteredPQR")
```

1.1 AlteredPQR_RB() function

The function takes as an input:
(i) a list of protein pairs that can form stable interactions, such as within protein complexes. An example dataset is avialable with the package: **int_pairs**, but a different dataset can be imported by the user in the object of the same name. As a default, we use uniprot protein identifiers.
(ii) a data matrix with quantiative proteomics measurements in which rows represent uniprot protein identifiers, and columns samples. To run the function, the user needs to define which columns contain reference measurements, and which test measurments that will be investigated for the presence of outliers. In the example dataset available with the package **quant_data_all**, reference measuremnts are in the columns 1:23. The remaining columns are investigated for the presence of outliers.

```
> cols_with_reference_data = 1:23
```

Now we are ready to run the function Altered_PQR_RB(). RB stands for 'reference based', as we need to have data for the background (reference) distribution of values. Here, we run it with the default parameters and save results in the object *RepresentativePairs*.

```
> RepresentativePairs = AlteredPQR_RB()
```

The object stores information on significant outliers. It lists protein pairs identified as outliers, the AlteredPQR score, samples in which their log ratio values were outliers compared to reference samples, direction in which their log ratio moved compared to reference samples and it provides information on whether single or both proteins contributed to the trend (i.e. outlier signal).

```
> head (RepresentativePairs)

         Protein_pair    Score
22      Q13105-Q9Y6K1 76.26833
1   A0A024QZQ1-I6L9H2 63.65863
7       P37275-Q92769 36.72746
9       Q9BPX3-Q9UBC3 27.34022
16      P23771-O15379 26.69760
13      Q12824-Q58EY4 24.89665
                                                       Significant_samples
22                                 Basal_7,Basal_12,Basal_13,Basal_17,Basal_18
1   Basal_1,Basal_2,Basal_3,Basal_5,Basal_6,Basal_7,Basal_11,Basal_16,Basal_18
7                          Basal_1,Basal_6,Basal_10,Basal_11,Basal_13,Basal_17
9                          Basal_3,Basal_6,Basal_12,Basal_16,Basal_11,Basal_15
16                          Basal_1,Basal_3,Basal_7,Basal_11,Basal_12,Basal_16
13                                 Basal_6,Basal_11,Basal_12,Basal_17,Basal_18
            Change Signal_contribution
22 Decreased_ratio       Q13105&Q9Y6K1
1  Decreased_ratio   A0A024QZQ1&I6L9H2
7  Decreased_ratio       P37275&Q92769
9  Both_directions       Q9BPX3&Q9UBC3
16 Decreased_ratio              P23771
13 Decreased_ratio       Q12824&Q58EY4
```

In addition, the function has the following parameters that can be modified by the user:
modif_z_score_threshold *(default 3.5)* - a threshold to start considering values as outliers
fraction_of_samples_threshold *(default 0.10)* - a fraction of test samples that need to be classifed as outlier for the protein pair to be listed
modif *(default 1)* - the higher the value it is, more strict it is for an individual protein to be considered to contribute to the outlier signal
filter_variable_in_ref_set *(default "NO")* - should protein pairs that have highly variable ratios in reference samples be excluded
write_table *(default "NO")* - should the results be saved in the text file
print_recomm *(default "NO")* - does the user wish to have information on the

distribution of modified z-scores in all investigated test samples and, based on this, recommended thresholds for the proteomics dataset provided by the user

1.2 CorShift() function

The function compares correlation values of studied protein pairs between two sample groups and it takes as an input:
(i) a list of protein pairs that can form stable interactions, such as within protein complexes. An example dataset is avialable with the package: **int_pairs**, but a different dataset can be imported by the user in the object of the same name. As a default, we use uniprot protein identifiers.
(ii) a data matrix with quantiative proteomics measurements in which rows represent uniprot protein identifiers, and columns samples. To run the function, the user needs to define which columns contain measurements for each of the two sample groups that will be compared. In the example dataset available with the package (**quant_data_all**), measurements for the group A are in the columns 1:23 and measurements for the group B are in the remaining 18 columns.

```
> samplesGroupA = 1:23
> samplesGroupB = (1+23):(23+18)
```

With a dataset on investigated protein pairs and a data matrix with quantitative proteomic measurments (example sets: **int_pairs** and **quant_data_all**), we are ready to run the function CorShift(). Here, we run it with the default parameters and save results in the object *Cor_results.*

```
> cor_results = CorShift()
```

The object lists protein pairs whose correlation values between the two sets of samples strongly differ. It provides information on the Pearson correlation scores and associated p-values in the two groups of samples, it summarizes how many samples from the two groups had measurements for both proteins and could be use for the analysis, and it calculates difference in correlation values between the two groups of samples.

```
> head (cor_results)
```

```
              Pearson_cor_samplesA cor_p_value Pearson_cor_samplesB cor_p_value
O14497-Q58EY4                 0.20      0.3689                 0.82      0.0000
Q09028-Q8N2Z9                -0.44      0.1042                 0.79      0.0021
O60264-Q13330                -0.21      0.3315                 0.76      0.0002
Q12824-Q16514                 0.01      0.9758                 0.73      0.0072
Q14839-Q13330                 0.09      0.6746                 0.73      0.0007
Q9BTC8-Q92769                -0.20      0.3630                 0.72      0.0008
              NumberOfSamplesA NumberOfSamplesB Correlation_shift
O14497-Q58EY4               23               18              0.62
Q09028-Q8N2Z9               15               12              1.23
O60264-Q13330               23               18              0.97
```

Q12824-Q16514	19	12	0.72
Q14839-Q13330	23	18	0.64
Q9BTC8-Q92769	23	18	0.92

In addition, the function has the following parameters that can be modified by the user:
shift_threshold *(default 0.6)* - a threshold to list pairs with a strong change in correlation values between the two sample groups
writeTable *(default F)* - if "writeTable = T", the resulting table will be saved as a text file protCorrelationShift.txt
min_cor_in_samples *(default 0.6)* - minimum Pearson correlation value the two proteins need to have in order to consider them as correlating in one of the sample groups
cor_signif *(default 0.01)* - p-value threshold for the Pearson correlation of the two proteins in order to consider them as significantly correlating in one of the sample groups
